# A systematic review of the evidence of the burden of bipolar disorder in Europe

**DOI:** 10.1186/1745-0179-5-3

**Published:** 2009-01-23

**Authors:** Liberty Fajutrao, Julie Locklear, Jennifer Priaulx, Anne Heyes

**Affiliations:** 1 Health Economics and Outcomes, AstraZeneca R&D, Södertälje, Sweden; 2 AstraZeneca Pharmaceuticals LP, Wilmington, DE, USA; 3 Adelphi Mill, Bollington, Cheshire, UK

## Abstract

**Background:**

Bipolar disorder is recognized as a major mental health issue, and its economic impact has been examined in the United States. However, there exists a general scarcity of published studies and lack of standardized data on the burden of the illness across European countries. In this systematic literature review, we highlight the epidemiological, clinical, and economic outcomes of bipolar disorder in Europe.

**Methods:**

A systematic review of publications from the last 10 years relating to the burden of bipolar disorder was conducted, including studies on epidemiology, patient-related issues, and costs.

**Results:**

Data from the UK, Germany, and Italy indicated a prevalence of bipolar disorder of ~1%, and a misdiagnosis rate of 70% from Spain. In one study, up to 75% of patients had at least one DSM-IV comorbidity, commonly anxiety disorders and substance/alcohol abuse. Attempted suicide rates varied between 21%–54%. In the UK, the estimated rate of premature mortality of patients with bipolar I disorder was 18%. The chronicity of bipolar disorder exerted a profound and debilitating effect on the patient. In Germany, 70% of patients were underemployed, and 72% received disability payments. In Italy, 63%–67% of patients were unemployed. In the UK, the annual costs of unemployment and suicide were £1510 million and £179 million, respectively, at 1999/2000 prices. The estimated UK national cost of bipolar disorder was £4.59 billion, with hospitalization during acute episodes representing the largest component.

**Conclusion:**

Bipolar disorder is a major and underestimated health problem in Europe. A number of issues impact on the economic burden of the disease, such as comorbidities, suicide, early death, unemployment or underemployment. Direct costs of bipolar disorder are mainly associated with hospitalization during acute episodes. Indirect costs are a major contributor to the overall economic burden but are not always recognized in research studies.

## Background

Our knowledge of the burden of bipolar disorders is based primarily on evidence from the United States. The lifetime prevalence of bipolar disorders in 2005 was estimated at 3.9% [1]. In a US population screen, 31.2% of individuals with positive screens on the Mood Disorder Questionnaire (MDQ) for bipolar I or II disorders, had previously received a diagnosis of unipolar depression [2]. Another US patient survey showed a 69% rate of misdiagnosis, primarily for MDD, among patients with bipolar disorder [3]. This high rate of misdiagnosis most likely makes these prevalence estimates of bipolar disorder conservative. Aside from the diagnosis itself, comorbidity, disability, unemployment, and suicide further add to the burden of the disease. Approximately 65% of people diagnosed with bipolar disorder have at least 1 other psychiatric ailment, with anxiety, substance abuse, and other mood disorders among the most common [4,5]. Other comorbidities including injury, poisoning, and mental and nervous system disorders are estimated to contribute a mean cost of US $2036 per patient [6]. Patients with bipolar disorder are also at higher risk of premature mortality with 1 in 5 patients committing suicide [7]. Total bipolar/affective disorder treatment and management costs in the US in 2003 were estimated at $US 30.4 to 43.7 billion (using 1990 values) [8]. The lifetime direct cost of bipolar disorder was estimated at $13 billion in 1998 [9] and the direct cost of managing a single patient was estimated at more than $3400/year in 1999 [10]. Carta and Angst [11] have argued that "the financial implications of bipolar disorder are only just beginning to be taken into account." They also highlight a lack of data on indirect costs, which are presumed to be significant. The recognition of bipolar disorder as a major mental health issue is best exemplified by STEP-BD (Systematic Treatment Enhancement Program for Bipolar Disorder), a current large scale study across the US.

Given the differences between US and European health care systems, clinical practices, health research policy-making, and payer environments, we undertook this literature review to determine the epidemiological, clinical, and economic burden of bipolar disease in Europe.

## Methodology

### Overview

We conducted a systematic review of the literature for publications on issues pertinent to the burden of bipolar disorder, including epidemiology (prevalence, incidence, misdiagnosis), patient-related issues (disability, unemployment, comorbidity, mortality, suicide, functional and symptomatic recovery), and costs (direct, indirect, intangible, and other). We also reviewed health technology assessments (HTA) and treatment guidelines.

### Literature search

For all areas of interest, we searched MEDLINE, EMBASE, and BIOSIS databases and conducted a general Google search. For cost issues, the National Health Service Economic Evaluation Database (NHS EED) was also used.

The search terms for all issues included "bipolar disorder" (MeSH or EMTREE heading), "Europe," "UK," "France," "Germany," "Italy," "Spain," and "Sweden" combined with terms for the issues of interest. These terms included "prevalence," "incidence," "epidemiology," "disab$," "incapacit$," "$employ$," "comorbid$," "concomitant condition," "function$" and "symptom$ recovery," "prognosis," "suicide," "case fatality," "mortality," "death," "misdiagnosis," "incorrect diagnosis," "undiagnosed," "under diagnosed," "cost," "economic or financial burden," "resource use," "hospitalization," "quality of life," "productivity loss," "absence," "off work or school," "crime," "criminal justice system" and "marriage."

We included observational and cost studies conducted during the past 10 years in the UK, France, Germany, Italy, Spain, and Sweden, with abstracts published in English or French since the reviewers were fluent in both languages. Reviews, case studies and studies relating to treatment outcome were excluded. During data extraction, quality was assessed using criteria based on Prins et al [12] for epidemiology, Downs and Black [13] for observational studies, and Cooper [14] for cost studies.

We also searched the Web sites of health technology assessment bodies such as NICE (National Institute for Health and Clinical Excellence], the SMC (Scottish Medicines Consortium], IQWIG (Institute for Quality and Efficiency in Healthcare), the LFN (Pharmaceutical Benefits Board) in Sweden, the Transparency Commission in France, the AIFA (Agenzia Italiana del Farmaco) in Italy, the NHS EED, the HEED (Health Economic Evaluation Database) and the CRD (Centre for Reviews and Dissemination) for HTAs, recommendations and treatment guidelines.

## Results

Overall, 7 epidemiology studies, 14 studies on other related issues in the bipolar population, 1 misdiagnosis study, and 4 cost studies met the predefined inclusion criteria (Table 1) [15-39].

**Table 1:** Studies included in the systematic review

Study	Country	Type of study	Study population	Sample size
Jacobi et al 2004 [15]	Germany	Prevalence survey	General	4181
Faravelli et al 2006 [16]	Italy	Prevalence survey	General	2363
Kennedy et al 2005 [17]	UK	Incidence survey	General	120,000
Kennedy et al 2004 [18]	UK	Incidence survey	General	120,000
Kennedy et al 2005 [19]	UK	Incidence survey	General	120,000
Lloyd et al 2005 [20]	UK	Incidence survey	General	1,631,462
Proctor et al 2004 [21]	UK	Incidence survey	General	249,203
Benedetti et al 2007 [22]	Italy	Cohort	BPI	72
Dutta et al 2007 [23]	UK	Cohort	BPI	120,000
Daban et al 2006 [24]	Spain	Cohort	BPI and BPII out-patients	300
Dittmann et al 2002 [25]	Germany	Cohort	Bipolar disorder [BPI, BPII, NOS] or schizoaffective disorder [bipolar type]	152
Brieger et al 2007 [26]	Germany	Cross-sectional	BPI inpatients	121
Henry et al 2003 [27]	France	Cross-sectional	Bipolar disorder	318
Schiavone et al 2004 [28]	Italy	Cross-sectional	Unipolar and bipolar disorder in-patients	300
Vieta et al 2001 [29]	Spain	Cross-sectional	Remitted BPI out-patients	129
Pini et al 2003 [30]	Italy	Cross-sectional	BPI in-patients	151
Benazzi 2006 [31]	Italy	Cross-sectional	BPII	374
Benazzi 2003 [32]	Italy	Cross-sectional	Unipolar and BPII outpatients presenting for major depressive episode	313
Benazzi 2004 [33]	Italy	Cross-sectional	BPII and unipolar MDD patients	73
Benazzi 2003 [34]	Italy	Cross-sectional	Depressive mixed state	433
Baca-Garcia et al 2007 [35]	UK	Cohort	Bipolar affective disorder adults	1153
Das Gupta & Guest 2002 [36]	UK	Prevalence-based cost study	Bipolar disorder	297,000
Finnern et al 2003 [37]	UK	Retrospective chart review	BPI and BPII	134
Zelicourt et al, 2003 [38]	France	Prevalence-based cost study	BPI	390,000
Olie & Levy 2002 [39]	France	Retrospective chart review	Hospitalization following manic episodes	137

BPI, bipolar I disorder; BPII, bipolar II disorder; MDD, major depressive disorder; NOS, not otherwise specified

### Epidemiology

Data from the UK, Germany, and Italy indicated that the prevalence of bipolar disorder in European countries was around 1%. Prevalence ranged between 0.6% to 1% in Germany and 0.8% in Italy. In the UK, the first-episode incidence over 2 years was estimated at 4% to 4.6% in 3 cities and at 0.01% over 3 years in another area [21]. In the cities, the age-related incidence of bipolar disorder varied between 1.7% and 6.2% [20]. Faravelli et al [16] found the prevalence of bipolar I and II disorder to be 0.5% and 0.4%, respectively.

There was strong evidence to show that bipolar disorder is often misdiagnosed and that consequently, prevalence and incidence rates are underestimated, as has been the case in the USA [2,3]. A Spanish study by Baca-Garcia et al [35] found that only 30% of patients with bipolar disorder were given a bipolar diagnosis on their first evaluation.

### Comorbidities

Fourteen studies showed evidence that patients with bipolar disorder suffer from comorbidities (Table 2) [15,16,22,24-34]. In particular, the evidence for concurrent mental disorders is strong compared with other conditions. Up to 75% of patients with any bipolar disorder had one or more *Diagnostic and Statistical Manual of Mental Disorders, Fourth Edition *(DSM-IV) comorbidities [15]. More specifically, anxiety disorders and substance and alcohol abuse appeared to be relatively common (16–70% for anxiety disorders and 21–34% for substance abuse disorders) among patients with bipolar disorder although this finding was dependent on study inclusion and exclusion criteria [22,26,27].

**Table 2:** Proportions of bipolar disorder patients with comorbidities

Study	Country	Outcome
Jacobi et al 2004 [15]	Germany	75% DSM-IV comorbidities
Faravelli et al 2006 [16]	Italy	28% GAD, 24% antisocial personality disorder, 16% PDA, 16% SP, 16% psychosis, 16% paranoid and 16% borderline personality disorder
Benedetti et al 2007 [22]	Italy	BPI comorbidities by gender F/M%: 67/70% anxiety disorder, 54/26% PD, 33/63% OCD, 12/33% history of substance disorder
Daban et al 2006 [24]	Spain	By manic/depressive onset: 22/27% Axis 1 comorbidity 17/24% Axis 2 comorbidity
Dittmann et al 2002 [25]	Germany	36% comorbid Axis I disorder
Brieger et al 2007 [26]	Germany	34% substance abuse disorders, 16% anxiety disorders and 22% personality disorders
Henry et al 2003 [27]	France	21% substance abuse, 24% anxiety disorder(s), 16% PDA, 11% phobia and 3% OCD
Schiavone et al 2004 [28]	Italy	Borderline personality disorder (41%), narcissistic disorder (21%), dependency disorder (13%) and histrionic (10%)
Vieta et al 2001 [29]	Spain	31% comorbid psychiatric diagnosis
Pini et al 2003 [30]	Italy	23% comorbid PD and 16% comorbid SP/OCD
Benazzi 2006 [31]	Italy	45(M)-59(F)% Axis I comorbidity
Benazzi 2003 [32]	Italy	54% Axis I comorbidity
Benazzi 2004 [33]	Italy	46% Axis I comorbidity
Benazzi 2003 [34]	Italy	54% Axis I comorbidity

DSM-IV, *Diagnostic and Statistical Manual of Mental Disorders, Fourth Edition*; GAD, general anxiety disorder; PD, panic disorder; PDA, panic disorder with agoraphobia; OCD, obsessive-compulsive disorder; SP, social phobia

### Mortality and suicide

Evidence for elevated suicide and premature mortality rates also adds to the economic burden of bipolar disease in Europe, primarily as a result of decreased productivity levels. However, the evidence for mortality is poor compared with that for suicide.

Dutta et al [23] reported death rates of 18% for bipolar I disorder patients in the UK and 3–7% suicide rate (although this was only 8 individuals in total). Attempted suicide rates varied between 21% and 54%. An Italian study by Benedetti and colleagues [22] reported that 22% of males and 54% of females with bipolar I disorder had a history of suicide attempts. In a French study by Henry et al [27], it was reported that 40% of bipolar patients had attempted suicide at least once. Vieta et al [29] found that 38% of bipolar patients with a comorbidity had attempted suicide compared with only 21% of patients without a comorbidity.

### Disability and unemployment

Dittmann and colleagues [25] found that only 30% of patients with bipolar disorder in Germany were employed full-time at a level that was appropriate for their qualifications. Other studies showed that between 52% and 59% of bipolar patients had an occupation [27,29], while an Italian study [40] found that 63% to 67% of patients with bipolar I disorder were unemployed. This is supported by evidence from a German study [26] which found that 72% of bipolar I patients received disability payments. Das Gupta and colleagues estimated absenteeism rates of 8% [36].

### Chronicity

Bipolar disorder is a long-term disease with many recurrences expected during the patient's lifetime. Demographic studies showed that on average, patients with bipolar disorder experienced an episode every few years. Vieta et al [29] reported that at a mean age of 40 years, the mean number of total episodes was 12 to 15. Brieger et al [26] reported an average of 9 episodes during a mean of 17 years of illness, while Daban [24] quoted an average of 11 to 15 episodes dependent on the nature of the onset episode. Dittmann and colleagues [25] found that in a 2.5-year follow-up period, only 27% of patients were free from symptoms, but 56% had a recurrence.

### Costs

There are very few published studies that help to measure the burden of disease in monetary terms, especially in the major European countries. However, despite the paucity of data, available evidence indicates that the cost-of-illness is high. Only 4 cost-of-illness studies were identified from the UK and France. The most extensive study [36], including both direct and indirect costs, estimated UK national costs of bipolar disorder at £4.59 billion (estimated 2007 value).

In this study [36], the costs associated with hospitalization of patients during acute episodes appear to represent the largest share of direct costs, at £69 million per year. This is supported by other studies, including 1 by Finnern et al [37] which anticipated a substantial role for hospitalization costs, especially for manic patients, who were hospitalized for an average of 65 days compared with 36 days for depressive patients. In France in 1999, total hospitalization costs were estimated at €2.75 billion [38].

However, when compared with the amalgamation of other long-term direct costs (including GP visits, prescriptions, tests, outpatient visits, psychiatric ward visits, community health teams, day hospitals, and special hospitals), other factors add up to £130 million per year [36]. Nonmedical long-term accommodation costs contribute £67.8 million and day care costs £18.1 million per year. Conversely, the cost of drugs was small relative to other direct costs, representing 4% to 6% of the total [36].

The key study from the UK [36] also included indirect costs of unemployment (£1510 million per year) and suicide (£179 million), adding to the burden of bipolar disease.

### HTA and guidelines

Limited evidence was available from HTA and guidelines. A systematic review of the clinical and cost-effectiveness of newer treatments for mania [41] found that the evidence was too limited and the quality too poor to analyze fully the comparative cost-effectiveness of treatments. A French cost-effectiveness analysis [42] showed similar limitations in quality of design and methodology.

A systematic review of the clinical and cost treatments for preventing relapse in bipolar disorder in 2007 [43] concluded that there was insufficient evidence to assess the impact of treatments on suicide or mortality. In general, HTA recommendations and guidelines in the UK and France focused on the treatment of individual manic or depressive episodes or prevention of recurrence rather than on general therapy for all phases of the disease.

### Quality

The epidemiology studies all scored between 10 and 14 out of 18 based on the Prins [12] criteria, which evaluate external (source population, eligibility criteria, response rate, nonresponders, study period, and population characteristics) and internal validity (prospective design, measurement instrument, disease definition, reporting of prevalence, and level of information).

The majority of the observational studies on other issues relating to the burden of bipolar disorder also scored between 10 and 14 out of 18 based on the Downs and Black [13] criteria. The criteria included reporting (outcomes, patients, loss-to-follow-up, interventions, confounders, findings, random variability of outcomes, and probabilities), external validity (whether study was representative and accounting for confounding, recruitment, sample size, and loss-to-follow up) and internal validity (bias of sub-group analysis, follow-up length, statistical analysis, and measures). Sample selection and its reporting were generally poor.

The cost-of-illness studies were evaluated in terms of the presence of 7 key criteria: viewpoint, population, medical/nonmedical costs, morbidity/mortality costs, discounting, incremental/attributable, and sensitivity analysis. One study [36] fulfilled 6 and 3 studies fulfilled 2 of the 7 criteria fully [37-39].

## Discussion

Our review indicates that the lifetime prevalence of bipolar disorder is approximately 1%. In the UK, the reported incidence ranged from 0.01% to 6.2%. This variation can be accounted for by several factors, including the time period over which data were collected, the location of mental health services, and the accuracy and consistency of the diagnosis and its coding in medical records. More recent data from the USA show a lifetime prevalence of 3.9%.

The reported prevalence of comorbidities ranged from 31% to 75% for comorbid psychiatric disorders and 13% to 28% for comorbid anxiety. The variation may be attributable to differences in evaluation, diagnosis, and reporting. There was insufficient information on case fatality, symptomatic recovery, functional recovery, and disability to make an analysis of the evidence. Even where evaluated, data on levels of employment were difficult to compare based on differing outcome measures. Evidence from the USA reinforces the important impact of bipolar disorder on suicide and quality of life [3,44-46] but there is a lack of similar evidence for Europe.

By its very nature, bipolar disorder represents a diagnostic challenge. In this review, only one study evaluated misdiagnosis rates [35]. A rate of 30% was reported by Baca-Garcia et al [35], which was lower than the USA values reported by Hirschfeld and colleagues, in which 31% of patients were misdiagnosed with MDD, 49% were undiagnosed with either MDD or bipolar disorder and 20% were correctly diagnosed [2]. An earlier study by Gonzalez-Pinto et al [47] reported that 31% of patients were misdiagnosed with schizophrenia or a psychotic disorder at onset.

Two French studies demonstrated high direct medical costs for mania episodes, including hospitalizations, regardless of study design [38,39] Reported costs ranged from €2.75 to £4.59 billion. UK and Dutch studies tried to demonstrate the indirect costs using NHS registry and epidemiological data, respectively [36,48,49]. The yearly UK estimate for indirect costs was £1510 million for unemployment while the Dutch estimate was US$1370 million including absence and inefficiency. Although the UK study was more comprehensive in its scope, it was conducted several years ago.

The true costs of bipolar disorder are likely to have been underestimated due to methodological limitations in the published articles. Limiting factors include incomplete coverage of costs, lack of clinical guidelines for diagnosis and treatment, reliance on public registries and literature sources for data analyses, and lack of knowledge of actual resource use in patients with bipolar disorder by stage, age group, country, and health care provider. Lost workdays due to sick leave, reduced working capacity, or death were not included in all cost studies.

Evidence of the indirect and social burden of bipolar disorder can be gleaned from general figures of mental health disorders. Statistics from the largest statutory health insurance fund in Germany (AOK-Bundesverband) showed that 24.9 individuals per 100,000 had at least 1 day of absenteeism for affective psychosis in 1999. The mean workday loss per individual was 46.8 days. A Spanish study (OFISALUD 1998, cited from Kleinman [8]) estimated that, in 1998, indirect costs represented almost 66% of the total costs of mental health disorders, with lost productivity accounting for 64.5% of these costs. Suicide and other causes related to mental disorders such as organic/alcoholic psychoses and motor vehicle accidents made up the remaining 35% [8].

A major issue related to the burden of bipolar disorder is the role of cognitive dysfunction as a key mediator of disability. Cognitive functioning has been shown to be associated with employment status among persons with bipolar disorder [50]. Studies have identified verbal memory [51], sustained attention [52], and executive function [53] as the specific areas of cognitive dysfunction implicated in bipolar disorder. The deficits have been noted in patients in the acutely manic [54] and acutely depressed [55] states. However, persistence of cognitive impairments has also been observed in the euthymic non-acute states of illness [51,56]. Therefore, people with bipolar disorder may continue to experience occupational and social disabilities even after they have experienced remission of symptoms. Given the need for full functional recovery and the consequences of long-term disability, cognitive functioning in bipolar disorder is an important topic worthy of further study.

It is evident that the economic burden of bipolar disorder is large. However, European evidence is scarce and patchy. Even in the USA, Carta and Angst [11] highlight that cost studies tend to focus on inpatient care, are influenced by late diagnosis, and lack indirect cost data. They cite ranges of $24 to 30 billion over a 1-year period. According to Diabetes UK, the economic burden of diabetes is £3.5 billion a year in the UK [57], which is less than the £4.6 billion per year for bipolar disorder estimated in a UK study [36]. Evidence from the Disease Control Priorities Project 2001, showed that annual losses in disability-adjusted life years (DALYs) were similar for schizophrenia and bipolar disorder [58]. However, schizophrenia appears to attract more interest and attention.

This review may have been subject to some degree of bias due to its focus on electronic databases such as MEDLINE, EMBASE, and BIOSIS and language selection criteria. Bias was minimized by including a "Google" search for references and checking reference lists in the selected studies.

## Conclusion

Data from the USA have identified bipolar disorder as a major mental health issue and an area of large unmet clinical need. There is a growing interest in the topic as exemplified by STEP-BD, which aims to improve knowledge and treatment of the disease. Conversely, in Europe, a paucity of evidence exists regarding the epidemiological, clinical, and economic burden of the disease.

Available data suggest that bipolar disorder is a major health problem. An estimated 1% of the population suffers from the disorder, although misdiagnosis likely underestimates the figure. Other issues may impact on the burden of the disease, such as comorbidities, suicide, early death, unemployment, or employment at a level below their qualifications.

The costs of bipolar disorder are high. Direct costs are mainly associated with hospitalizations, GP or outpatient visits, and community care. Indirect costs (lost productivity in particular) are a major contributor to the burden of disease but are not always recognized in research studies. The burden of bipolar disorder to patients, health care systems, and society in Europe is large and to date has been under-recognized. The costs are mainly due to long-term indirect costs and not only to hospitalization during acute episodes. Drug costs are relatively small by comparison. Therapy that is effective in all phases of the disease and to which patients adhere is essential for effective management.

In addition, the methodology used in the studies included in this systematic review was generally of limited depth. While epidemiological studies are often based on retrospective reviews and nonrepresentative samples, cost studies can vary widely and fail to include all costs associated with bipolar disorder.

The current state of evidence suggests that bipolar disorder is not considered to be an important enough health issue in Europe to warrant research. Despite the methodological challenges pertinent to bipolar disorder in particular, and to mental health in general, more research and sustained interest in the disease area is needed.

## Competing interests

LF and JL are employees of AstraZeneca. AH is an employee of MAPI Values and JP is a former employee of MAPI Values.

## Authors' contributions

LF and JL conceived of the study and participated in its design and interpretation. JP and AH also participated in the study design, coordination, and analysis. All authors read and approved the final manuscript.
